# Prolactin concentration in various stages of endometriosis in infertile women

**DOI:** 10.5935/1518-0557.20190020

**Published:** 2019

**Authors:** Parvaneh Mirabi, Seyede Hanie Alamolhoda, Masoumeh Golsorkhtabaramiri, Mahshid Namdari, Sedighe Esmaeilzadeh

**Affiliations:** 1 Infertility and Reproductive Health Research Center, Health Research Institute, Babol University of Medical Science, Babol, Iran; 2 Midwifery and Reproductive Health Research Center, Shahid Beheshti University of Medical Science, Tehran, Iran; 3 Department of Community Oral Health, School of Dentistry, Shahid Beheshti University of Medical Science, Tehran, Iran

**Keywords:** hyperprolactinemia, endometriosis, infertility

## Abstract

**Objective:**

The relation between excessive prolactin and endometriosis-related infertility is debatable. Anovulation or defective luteal phase occurs frequently due to hyperprolactinemia in subfertile women. In this investigation, we evaluated the association between serum prolactin levels and the severity of endometriosis.

**Methods:**

This retrospective cohort study carried out at the Babol Infertility Research Center looked into the baseline serum prolactin levels of 114 infertile women with endometriosis and compared them to the levels seen in 101 infertile women without endometriosis (controls). Statistical analysis included independent t-test, chi-square, Welch test and ROC curve analysis.

**Results:**

Infertile women with endometriosis had significantly higher serum prolactin levels than infertile women without endometriosis (*p*=0.003). A significant difference was detected between controls and individuals with endometriosis stages III/IV (*p*-value=0.009). Prolactin was found to have diagnostic value to detect endometriosis stages III/IV vs. stages I/II in AUC=0.65, 95% CI (0.55, 0.76). Prolactin values with a cut off set at 20.08 ng/mL had a sensitivity of 0.74 and specificity of 0.54 in detecting disease stages III/IV vs. I/II. The prognostic capability of prolactin in detecting endometriosis in cases vs. controls by ROC curve analysis had an AUC=+0.67, 95% CI (0.60, 0.74). Prolactin values with a cut off set at 17.5 ng/mL had a sensitivity of 0.64 and specificity of 0.63 in segregating subjects with and without endometriosis.

**Conclusion:**

Higher prolactin levels were observed in infertile women with more severe endometriosis when compared to infertile women without endometriosis. Prolactin levels act as a probable prognostic biomarker to detect endometriosis stages III/IV vs. I/II and segregate infertile women with endometriosis from subjects without endometriosis.

## INTRODUCTION

Endometriosis is a gynecological disorder affecting the wellbeing of 5%-15% of women of reproductive age, with a prevalence of 5%-50% in infertile women and 32% in women with chronic pelvic pain (Bellelis *et al*., 2014). Studies showed that 30% to 50% of the women with endometriosis are infertile (Zhang *et al*., 2014; Zhu *et al*., 2014). Endometriosis is the presence of endometrial tissue (glandular epithelium and stroma) outside the normal location (Abu Hashim, 2014). An estimated seven million women have endometriosis in the USA, and the disease ranks as one of the main causes for gyneco logical hospitalization in industrialized nations. Gao *et al.* reviewed the direct medical and nonmedical burden associated with endometriosis. The authors suggested that endometriosis places a considerable burden on patients and society (Bellelis *et al*., 2014; Gao *et al*., 2006).

According to the definition of the American Society of Reproductive Medicine (ASRM), endometriosis can be categorized into four stages: stage I (minimal), stage II (mild), stage III (moderate), and stage IV (severe) (Pacchiarotti *et al*., 2014). More advanced stages may be deeply invasive and present as endometrioma (Avcioğlu *et al*., 2014). Nearly a third (32%) of the patients with endometriosis have moderate to severe disease, while 58% have minimal or mild endometriosis. The pathogenesis of mild/minimal endometriosis with infertility is unclear (Zhu *et al*., 2014). Diagnostic laparoscopy, with or without biopsy for histological diagnosis, is the most common procedure used to diagnose and remove mild to moderate endometriosis. This method is considered the gold standard among scoring systems available for determining disease severity (Marchino *et al*., 2005).

Investigators have suggested that women with mild to moderate endometriosis have a higher incidence of endocrine abnormalities, anovulation, and hyperprolactinemia. However, other well-organized prospective studies have found most of these factors to be either normal or lacking in clinical significance (Gardner *et al*., 2017). Nevertheless, several clinical and experimental reports have suggested a relationship between endometriosis and its progression with hyperprolactinemia. There is controversy as to whether abnormal prolactin secretion is directly involved in infertility in patients with endometriosis (Gardner *et al*., 2017; Esmaeilzadeh *et al*., 2015).

The real mechanisms of infertility associated with endometriosis in patients with hyperprolactinemia have not been entirely clarified. Regardless of the interventional role of hyperprolactinemia in the endocrine pattern of infertility, it probably impairs luteinizing hormone (LH) pulsation and induces infertility through ovulation failure, luteinized unruptured follicle (LUF) syndrome or poor endometrial response to estrogen (Wang *et al*., 2009). The studies that suggested a relationship between endometriosis and abnormal prolactin secretion are limited in number, and their results are controversial. In addition, far too little attention has been given to studies comparing prolactin levels in various stages of endometriosis.

In a previous study (Esmaeilzadeh *et al*., 2015) we found a relationship between endometriosis and prolactin levels; in this study, we looked into whether hyperprolactinemia is a probable prognostic biomarker to detect the severity of the endometriosis (minimal to severe) by analyzing further samples.

## MATERIAL AND METHODS

This is a retrospective cohort study. The data sets used herein were extracted from the medical records of patients seen at the Infertility and Reproductive Health Research Center at Babol University of Medical Science from January 2015 to September 2016. The data collected included age, reasons and duration of infertility, stages of endometriosis, serum prolactin (PRL) levels, and ultrasound/laparoscopy findings.

Serum PRL was measured with DiaSorin kits manufactured in Spain and with the aid of a LIAISON system using chemiluminescence technology (CLIA). PRL secretion was deemed normal when baseline serum levels were 25ng/ml or lower at least two hours after waking up in the morning (Melmed *et al*., 2011). Patients categorized as having hyperprolactinemia had to have two prolactin level readings ≥25 ng/ml on the second or third day in two consecutive periods. Also, a patient was considered in the normal prolactin group if the level of prolactin was normal at once. Since our patients had infertility and irregular menstrual periods, endocrine tests were run to exclude other potential ovarian endocrine defects that might have affected their status of infertility associated with endometriosis. The research project was approved by the Ethics Committee of the Babol University of Medical Science and written consent was obtained from all participants.

### Participants

The group with endometriosis contained infertile patients with endometriosis confirmed by laparoscopic examination. They were further segregated into two subgroups, stage I/II endometriosis and stage III/IV endometriosis, and all patients with endometriosis were scored according to the World Endometriosis Society consensus on the classification of endometriosis (Johnson *et al*., 2017). The infertile patients in the group they were compared against underwent laparoscopic examination and had no signs of endometriosis. 

Excluded patients were older than 40 years, had diseases such as thyroid dysfunction or renal disease or were taking drugs that caused hyperprolactinemia, or were in non-fasting conditions, exercised excessively, had trauma, renal disease or inadequate data for analysis. 

### Statistical analysis

Statistical analysis was performed on SPSS 19.0. The data were tested for normality with the Kolmogorov-Smirnov test and were presented as mean values ± (SD) or percentages when appropriate. The independent t-test was used to compare between baseline PRL levels of the two groups; the chi-squared test was used to determine the relationships between categorical variables; and the Mann-Whitney Test was used to compare the sample mean values coming from one same group. Linear regression and logistic regression were used to determine the association between prolactin levels and stages of endometriosis. All tests were two-tailed and significant differences had a *p*-value of less than 0.05.

## RESULTS

One hundred and twenty-three women were diagnosed with endometriosis. Nine were excluded for different reasons (three were on pills for thyroid disorder; two were not accessible; and three chose not to join the study). Of the 114 patients with endometriosis enrolled in the study, 37 (32.4%) had disease stages I/II (5 with stage I, 32 with stage II) and 77 (67.5%) had endometriosis stages III/IV (38 with stage III, 39 with stage IV). One hundred and one patients were included in the control group. No one from the control group was excluded. There were no statistically significant differences in age, level of education, body mass index or primary infertility between the endometriosis and control groups (Table 1).

**Table 1:** Study population

Characteristics	Endometriosis (n=114)	Control (n=110)	*p*-value
Age (years) (Mean)*	31.06±5.22	29.49±6.40	0.05 ^a^
BMI (kg/m^2^) (Mean)*	25.77±4.33	26.60±4.35	0.16 ^a^
Duration of infertility (years) (Mean)**	5.04±5.67	3.52±2.61	0.01 ^a^
Education (n)** (n, %)			
Elementary education	11 (9.6)	22 (21.7)	0.53 ^b^
High school	68 (59.6)	63 (62.3)	
College education	35 (30.7)	16 (15.8)	
Primary infertility (n, %)	88 (77.2)	70 (69.3)	0.09 ^b^
Dyspareunia (n, %)			
Deep	55 (48.2)	23 (22.8)	0.000 ^b^
Superficial	8 (7)	3 (3)	
No Dyspareunia	51 (44.7)	75 (74.3)	

a Student’s *t*-test

b χ2

* Data presented as mean values ± (SD).

** Data presented as n (%)

The hormonal assay results of both groups are presented in Table 2. The mean PRL level was 17.88±12.81 ng/mL in the control group (Table 2); 23.42±34.05 ng/mL in the group with disease stages I and II; and 31.62±38.09 ng/mL in the group with disease stages III and IV. Serum prolactin levels were significantly higher among infertile women with endometriosis than in infertile women without endometriosis (*p*=0.003) (Table 2). Welch’s test revealed significant differences between the three groups (*p*=0.018). Tamhane’s multiple comparison test revealed a significant difference between controls and individuals with disease stages III/IV (*p*=0.009). The related *p*-value indicates that there is a 0.009 probability that chance produced in the relation of endometriosis stages III/IV and prolactin value, however the calculated effect size of the study with 80% confidence showed -0.55 with CI (-0.74 - -0.35).

**Table 2:** Hormonal assay results of women with and without endometriosis

Hormone	Endometriosis (n=114)	Control (n=110)	*p*-value
FSH (mIU/L)	6.52±3.25	7.14±3	0.17
LH (mIU/L)	5.61±3.86	5.56±3.15	0.92
TSH (mIU/L)	3.12±9.84	3.04±6.96	0.95
PRL (ng/mL)	28.96±3.88	17.88±2.81	0.003

Mean values ± SD

**Figure 1:**
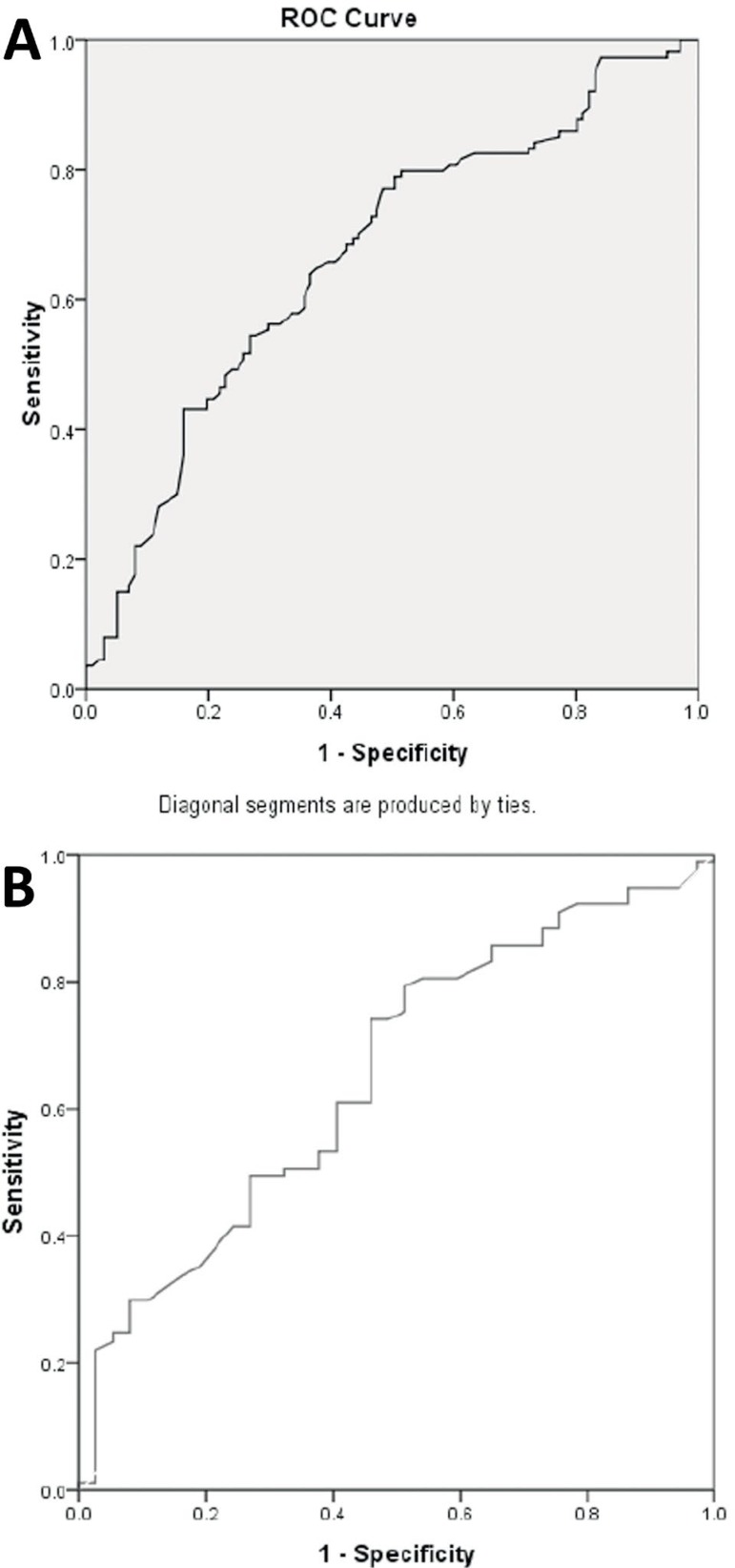
A. ROC Curve to assess the diagnostic capability of prolactin in differentiating between patients with and without endometriosis. B. ROC Curve to assess the diagnostic capability of prolactin in differentiating patients with endometriosis stages III/IV from patients with endometriosis stages I/II

The differences between controls vs. subjects with disease stages I/II and stages I/II vs. stages III/IV were not significant (*p*=0.71, *p*=0.58). The calculation of the effect size of stages I/II vs. stages III/IV showed that the strength of association was -0.23 with 80% CI (-0.42 - -0.03). The prognostic capability of prolactin in detecting endometriosis stages III/ IV vs. stages I/II was analyzed by ROC curve analysis (Figure 1). Prognostic capability was achieved with an AUC=0.65, 95% CI (0.55, 0.76). Prolactin values with a cut off set at 20.08 ng/mL had a sensitivity of 0.61 and specificity of 0.60 to detect endometriosis stages III/IV vs. I/II. In addition, the prognostic capability of prolactin in segregating endometriosis cases from controls was identified by ROC curve analysis with an AUC=+0.67, 95% CI (0.60, 0.74). Prolactin levels of 17.5 ng/mL had a sensitivity of 0.64 and specificity of 0.63 to differentiate endometriosis cases from controls.

## DISCUSSION

Our previous study revealed that infertile patients with endometriosis had hyperprolactinemia (Esmaeilzadeh *et al*., 2015). In the present study, the most striking result to emerge from our data is that we found a good prognostic capability of prolactin in detecting patient with endometriosis stages III/IV vs. stages I/II. A clinical implication of these findings is proposing hyperprolactinemia (Prolactin values ≥20.08 ng/mL) as a probable prognostic biomarker for infertile women with endometriosis stages III/IV vs. I/II. We were unable to find a study that reported a cutoff value for prolactin in women with endometriosis stages III/IV vs. I/II. Moreover, the present study demonstrated that the serum prolactin levels of infertile women with endometriosis stages III/IV were significantly higher than the levels seen in infertile women without endometriosis. This finding is supported by prior studies (Barbosa *et al*., 2014; Lima *et al*., 2006; Gregoriou *et al*., 1999; Cunha-Filho *et al*., 2001). By investigating a larger series of patients when compared to the previous study, we also found a cutoff value that increases the chance of telling individuals with endometriosis from controls (PRL >17.5ng/mL). The strength of the association was clearly confirmed in effect size analysis.

This finding agrees the observations made by Bilibio *et al*. (2014), which showed that serum prolactin could also be applied as a test for peritoneal endometriosis. Some authors described that increases in baseline serum prolactin might have a causative role in infertility affecting patients with severe endometriosis (Lima *et al*., 2006). It is unclear whether increased prolactin levels are the cause or consequence of endometriosis. In fact, estradiol stimulates prolactin receptors in the uterus. In the presence of ectopic endometriotic tissues, prolactin receptors are overly induced. Inversely, increased baseline serum prolactin reduces estrogen activity (Gunin *et al*., 2002). Lowering prolactin secretion reestablishes functional ovulation and improves endometrial development. Other authors have supported the use of prolactin inhibitors such as dopaminergic drugs to favor fecundity (Weil, 1986; Crosignani, 2012). Others reported the use of antiestrogens such as Tamoxifen to decrease estrogen-stimulated prolactin levels in hyperprolactinemic rats (Spritzer *et al*., 1996; Aquino *et al*., 2016). Future studies might provide a better understanding of the role of estrogen-dependent medicine on the progression of endometriosis.

It was somewhat surprising to see that the prolactin level difference observed between individuals with disease stages I/II and stages III/IV in this study was not significant. The related effect size showed that the strength of association was poor. It seems plausible that the related non-significant *p*-value might be due to the inadequate size of the samples of individuals with different stages of endometriosis. The authors wondered whether the association might have been stronger if a larger sample had been selected. In future studies, it is suggested that the associations between the various stages of endometriosis be investigated with larger samples of individuals with different stages of endometriosis.

Unfortunately, we had trouble selecting individuals with endometriosis stage I. Endometriosis is often undiagnosed or misdiagnosed in affected women who come to the clinic looking for care. In other words, there is a time gap between the onset of symptoms and the diagnosis of endometriosis. Barbieri (2017) reported a gap of more than eight years between the age of pelvic symptom onset and the age of diagnosis. Possible explanations for this gap include lack of knowledge, variations in the manifestations of endometriosis, overlapping symptoms with other pelvic diseases, unwillingness to undergo laparoscopy in early stage disease, concerns around nonsurgical methods, and the costs associated with endometriosis care. Nevertheless, most patients with minimal or mild endometriosis have normal function and do not have to see a physician for early diagnosis. Most return for care after the disease has progressed. At the same time, affected women are exposed to higher blood prolactin levels for years. Even when not associated with endometriosis, elevated prolactin levels produce devastating short- and long-term effects and dramatically interfere with the reproductive and endocrine systems of patients who are not treated in a timely manner (Ballard *et al*., 2008). It is likely that improvements to endometriosis care might shorten the time gap until diagnosis, particularly in early-stage disease (Weintraub, 2016).

## CONCLUSION

Infertile women with more advanced endometriosis have higher prolactin levels than infertile women without endometriosis. Prolactin is a probable prognostic biomarker to detect endometriosis stages III/IV vs. I/II and to differentiate infertile women with endometriosis from infertile women without the condition. Prolactin levels might be helpful in the detection of endometriosis.
